# Causal associations between sleep traits and temporomandibular disorders: a bidirectional mendelian randomization analysis

**DOI:** 10.3389/fgene.2024.1429190

**Published:** 2024-07-24

**Authors:** Lihan Xu, Haojing Zhou

**Affiliations:** ^1^ School of Stomatology, Hangzhou Normal University, Hangzhou, Zhejiang, China; ^2^ The First School of Clinical Medicine, Zhejiang Chinese Medical University, Hangzhou, Zhejiang, China

**Keywords:** insomnia, mendelian randomization, sleep, snoring, temporomandibular disorders

## Abstract

**Objectives:**

This study aims to investigate the relationship between five sleep traits (insomnia, sleep duration, getting up in morning, snoring, and daytime nap) and temporomandibular disorders (TMD) using bi-directional Mendelian randomization.

**Methods:**

The bi-directional Mendelian randomization study was conducted in two stages. Initially, sleep traits were examined as exposures while TMD was evaluated as an outcome, whereas the second step was reversed. The inverse variance weighted (IVW) method and other Mendelian randomization methods were used for analysis. Furthermore, we performed the MR-Egger intercept, MR-PRESSO, Cochran’s Q test, and “Leave-one-out” to assess the levels of pleiotropy and heterogeneity.

**Results:**

The IVW method indicates that getting up in the morning reduces the risk of developing TMD (OR = 0.50, 95% CI 0.30–0.81, *p* = 0.005), while insomnia may increase the risk of TMD (OR = 2.05, 95% CI 1.10–3.85, *p* = 0.025). However, other sleep traits are not associated with the risk of TMD, and having TMD does not alter an individual’s sleep traits. After removing outliers, the results remained robust, with no pleiotropy detected.

**Conclusion:**

Genetically determined difficulty in getting up in the morning and insomnia can increase the risk of TMD. By optimizing sleep, the risk of developing TMD can be reduced. This underscores the importance of sleep in preventing TMD.

## 1 Introduction

Temporomandibular disorders (TMD) is a complex clinical condition characterized by a diverse array of symptoms, including restricted lower jaw movement, muscle pain, discomfort in the temporomandibular joint, joint sounds, systemic myofascial pain, and irregularities and limitations in jaw movement (Durham et al., 2015). Epidemiological research has revealed that TMD affects 5%–12% of adults, with an annual financial cost exceeding 100 billion dollars in the United States alone (Jin et al., 2016; Lee et al., 2021). Therefore, exploring the etiology of TMD and its prevention is crucial.

The etiology of TMD is multifactorial, yet still remains unclear. Commonly cited factors include biologic, environmental, social, emotional, and cognitive triggers (Gauer and Semidey, 2015). Increasingly, research suggests a link between sleep and TMD (Mun et al., 2022; Pala Mendes et al., 2022). Individuals with TMD often experience symptoms such as poor sleep quality and difficulty falling asleep. Furthermore, it has been associated with many oral diseases such as periodontitis and tooth loss (Han and Park, 2018; Alqaderi et al., 2020). Sleep plays a crucial role in maintaining human health, impacting various vital physiological functions. However, current studies on sleep and TMD are primarily cross-sectional and thus are unable to establish causal relationships.

Mendelian randomization (MR) is an epidemiological method that utilizes genetic variants strongly associated with an exposure as instrumental variables (IVs) to estimate the causal effect of the exposure on an outcome (Smith and Ebrahim, 2003; Lawlor et al., 2008). Conventional analyses may face difficulties in establishing causality due to potential biases from confounders, reverse causation, and measurement error, even when a statistically significant association between the exposure and outcome is present. In contrast, MR offers an alternative approach for exploring causality. Building on this, bidirectional MR aims to explore the reciprocal causal relationships between two variables. First, it analyzes the influence of exposure factors on outcome variables. Then, it reverses exposure and outcome variables to investigate reverse causal relationships.

To our best knowledge, there has been no bidirectional MR study investigating the association between sleep traits and TMD. Additionally, no randomized controlled trial has been specifically designed to assess the bidirectional relationship. Hence, we conducted a bidirectional MR study to explore the association between sleep traits and TMD, aiming to determine whether sleep traits contribute to the development of TMD and if TMD influences alterations in sleep traits. We hypothesize a causal relationship between sleep traits and TMD, suggesting that poor sleep habits may increase the risk of TMD, and TMD itself may also lead to changes in sleep traits.

## 2 Methods

### 2.1 Study design

**FIGURE 1:**
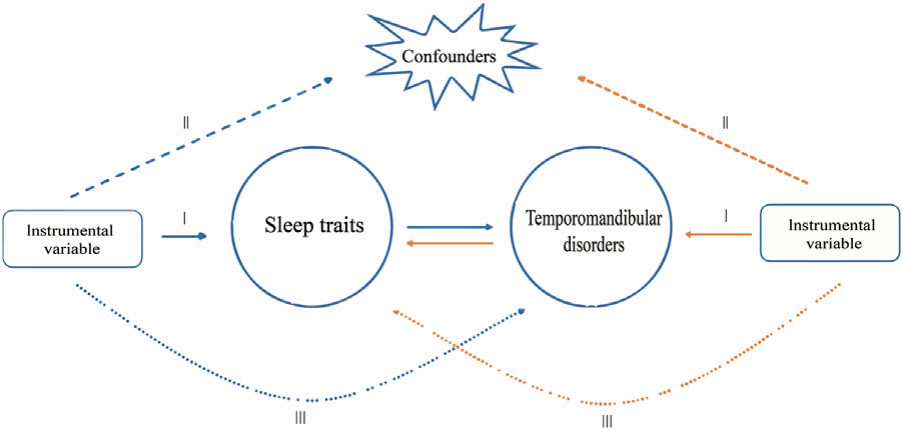
Study design overview. The blue arrows represent the forward study whereas the yellow arrows represent the reverse study.

In this study, a MR analysis was performed using Single Nucleotide Polymorphisms (SNPs) derived from summary data of a genome-wide association study (GWAS). Three assumptions must be met for SNPs selected as IVs in MR analysis (Sekula et al., 2016): firstly, chosen SNPs as IVs must be closely linked to exposures; secondly, IVs must be free of confounders; and lastly, IVs should affect the outcome exclusively through exposure, without a direct effect. The study consisted of two stages. In the initial stage, we investigated the causal relationship between sleep traits and TMD. Subsequently, in the second stage, we evaluated the association between genetic characteristics of TMD and sleep traits (Figure 1).

### 2.2 GWAS data for sleep traits and TMD

We utilized GWAS summary-level data on five sleep traits (insomnia, sleep duration, getting up in morning, snoring, and daytime nap) sourced from extensive genetic consortia accessible via the PubMed and IEU databases (https://gwas.mrcieu.ac.uk).

Insomnia, a prevalent sleep disorder, is characterized by difficulty either falling asleep or remaining asleep for the desired duration. Genetic associations with insomnia were identified through a GWAS conducted among 462,341 participants in the United Kingdom Biobank. Insomnia was evaluated using the query: " Do you have trouble falling asleep at night or do you wake up in the middle of the night? If this varies a lot, answer this question in relation to the last 4weeks” This assessment was part of the larger data collection efforts within the United Kingdom Biobank.

Genetic association data for sleep duration were obtained from the United Kingdom Biobank, comprising 460,099 adults of European descent. Respondents answered the question, “About how many hours of sleep do you get every 24 h (please include naps)," revealing a mean sleep duration of approximately 7 h. Moreover, over 80% of the samples fall within the range of 6–8 h.

Genetic association data regarding getting up in morning were collected from the United Kingdom Biobank, encompassing 461,658 participants. This data was obtained through the question, “On an average day, how easy do you find getting up in the morning?"

Genetic association data regarding snoring were collected from the United Kingdom Biobank, encompassing 430,438 participants (270,007 cases and 160,431 controls). The assessment of snoring involved asking participants the question, “Does your partner or a close relative or friend complain about your snoring?” Participants could respond with a “yes” or a “no”.

Daytime nap refers to a short period of sleep, typically taken during the daytime as a supplement to the usual nocturnal sleep period. Summary data from GWAS involving 462,400 individuals in the EBI database reveal a genetic association with daytime napping. This data was obtained by asking participants, “Do you have a nap during the day?”

Due to the predominance of GWAS data on sleep traits originating from the United Kingdom Biobank, to avoid overlap, the GWAS for TMD was drawn from the recent FinnGen research project (Kurki et al., 2023). This project comprised 6,314 cases and 222,498 controls. The FinnGen research project aims to elucidate genotype-phenotype correlations within the Finnish population. Cases of TMD were defined using the International Classification of Diseases, Tenth Revision (ICD-10) codes K07.60, K07.61, K07.62, and K07.63 (https://risteys.finregistry.fi/endpoints/TEMPOROMANDIB), excluding individuals with painful conditions affecting the limbs, back, neck, and abdomen.

### 2.3 Selection of IVs

To enhance the robustness of the MR analysis results, we meticulously screened eligible SNPs as IVs and performed MR analysis on exposures and outcomes (Burgess et al., 2017). First, we identified SNPs linked to exposures (*p* < 5 × 10^−8^). Second, to avoid biased results due to strong linkage disequilibrium among the selected SNPs, we performed a clumping process (*r* ^2^ < 0.001, clumping distance = 10,000 kb) to eliminate linkage disequilibrium between the included IVs. Third, SNPs associated with outcomes (*p* < 5 × 10^−8^) were excluded from the IVs. Fourth, to ensure a strong association with exposure, we chose SNPs with an F statistic >10 as IVs. F statistics were calculated using the formula: F = *R* ^2^/(1-R^2^) ×(N-K-1)/K. *R* ^2^ was calculated using the formula: *R* ^2^ = 2×MAF×(1-MAF) ×Beta^2^. Fifth, palindromic SNPs with intermediate allele frequencies were excluded to ensure that the influence of SNPs on exposures aligned with the same allele influencing outcomes.

Moreover, SNPs associated with TMD at a genome-wide significance level of *p* < 5 × 10^−6^ were chosen to ensure an adequate number of IVs.

### 2.4 Statistical analysis

All data analyses were performed using the TwoSampleMR packages in R version 4.3.1. First, the causal effect was primarily estimated using the standard inverse variance weighted (IVW) method (Burgess et al., 2015). Additionally, MR-Egger and weighted median (WM) analyses were conducted as supplementary analyses. Second, Cochran’s Q statistic from the MR-IVW method and Rucker’s Q statistic from the MR Egger method were employed to assess the heterogeneity in MR analysis (Cohen et al., 2015). Third, the intercept test of MR Egger and the global test of MR pleiotropy residual sum and outlier (MR-PRESSO) were employed to detect horizontal pleiotropy. Fourth, the distortion test in MR-PRESSO analysis was utilized to identify whether the MR analysis results were influenced by outliers. If outliers were identified, a second round of MR analysis was conducted after their removal. Fifth, the robustness of MR analysis results was evaluated using the leave-one-out test (Verbanck et al., 2018). To address multiple testing, we applied a Bonferroni-corrected threshold of *P*-value <0.01 (equivalent to *P* < 0.05/5). *P*-values falling between 0.01 and 0.05 were deemed suggestive of associations.

## 3 Results

### 3.1 Selection of IVs

After screening for SNPs associated with the exposures and removing linkage disequilibrium, we identified 42, 70, 76, 44, 98, and 15 SNPs for Insomnia, sleep duration, getting up in the morning, snoring, daytime nap, and TMD, respectively. With the exception of snoring, the F-statistic value for each selected IV exceeded 10, meeting the assumption of strong relevance for MR studies. For snoring, 34 SNPs with F-statistic values less than 10 were removed, leaving 10 SNPs. Supplementary Material S1 provide detailed information about all SNPs.

When sleep traits were utilized as exposure variables, for insomnia, three palindromic SNPs were identified: rs2644128, rs2803296, rs8180817. Eventually, 39 SNPs were chosen for subsequent analyses. Regarding sleep duration, 1 SNP was unavailable in the summary data for TMD, and 2 palindromic SNPs were found: rs17732997, rs2186122. Eventually, 67 SNPs were selected for subsequent analyses. As for getting up in the morning, 2 SNPs were not present in the summary data for TMD, and 2 palindromic SNPs were observed: rs11629621, rs1914397. Eventually, 72 SNPs were chosen for subsequent analyses. No palindromic SNPs were identified for snoring. Concerning daytime nap, 6 SNPs were absent in the summary data for TMD, and 5 palindromic SNPs were identified: rs11688767, rs13023587, rs1856502, rs1931175, rs285815. Eventually, 87 SNPs were selected for subsequent analyses.

When TMD was utilized as the exposure variable, only 1 palindromic SNP (rs1811546) was identified. Eventually, 14 SNPs were chosen for subsequent analyses.

### 3.2 Causal effect of sleep traits on TMD

Table 1 displays the results of MR analysis with sleep traits as exposure variables and TMD as outcome variables.

**TABLE 1:** The causal effects of sleep traits on temporomandibular disorders.

Exposure	Outcome	nSNP	Methods	OR (95% CI)	*P*-value	Heterogeneity		Pleiotropy P-val
						MR-Egger Q_pval	IVW Q_pval	
Insomnia	TMD	39	IVW	2.05 (1.10–3.85)	**0.025**	0.193	0.145	0.120
		39	MR Egger	0.51 (0.08–3.16)	0.477			
		39	WM	2.18 (0.91–5.24)	0.081			
Sleep duration	TMD	67	IVW	0.84 (0.52–1.37)	0.490	**0.038**	**0.044**	0.650
		67	MR Egger	1.30 (0.19–9.10)	0.790			
		67	WM	1.06 (0.55–2.04)	0.860			
Getting up in morning	TMD	72	IVW	0.50 (0.30–0.81)	**0.005**	**0.008**	**0.009**	0.762
		72	MR Egger	0.66 (0.09–4.64)	0.681			
		72	WM	0.52 (0.29–0.96)	**0.035**			
Snoring	TMD	10	IVW	3.47 (0.73–16.37)	0.116	0.364	0.451	0.768
		10	MR Egger	0.55 (0.01–88637.23)	0.924			
		10	WM	0.68 (0.08–5.64)	0.724			
Daytime nap	TMD	87	IVW	1.08 (0.63–1.85)	0.787	**0.014**	**0.018**	0.664
		87	MR Egger	1.64 (0.23–11.76)	0.623			
		87	WM	1.28 (0.65–2.55)	0.477			

TMD: temporomandibular disorders; IVW: inverse variance weighted; WM: weighted median.

The bold values represent *P*-value <0.05.

**FIGURE 2:**
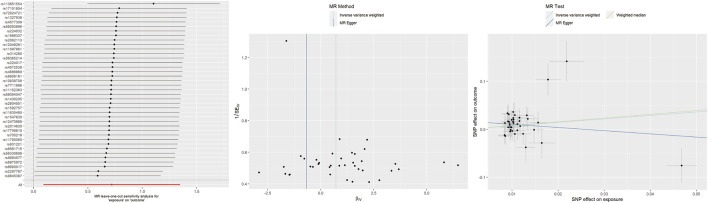
Leave-one-out plots, funnel plots, and scatter plots for insomnia as exposure and TMD as outcome.

For insomnia, IVW analysis indicated that genetically determined insomnia patients had an increased risk of TMD (OR = 2.05, 95% CI 1.10–3.85, *p* = 0.025). However, causal estimates from MR-Egger and WM methods did not reach statistical significance (MR-Egger: OR = 0.51, 95% CI 0.08–3.16, *p* = 0.477; WM: OR = 2.18, 95% CI 0.91–5.14, *p* = 0.081). No evidence of heterogeneity or horizontal pleiotropy was found. Leave-one-out analysis indicated that excluding any individual SNP did not significantly alter the overall evaluation conducted by the IVW method (Figure 2).

Regarding sleep duration, all three analysis methods indicated that genetically determined sleep duration was not associated with the risk of TMD (IVW: OR = 0.84, 95% CI 0.52–1.37, *p* = 0.490; MR-Egger: OR = 1.30, 95% CI 0.19–9.10, *p* = 0.790; WM: OR = 1.06, 95% CI 0.55–2.04, *p* = 0.860). Heterogeneity was observed following the heterogeneity test. Upon removing the source of heterogeneity (rs62444917), sleep duration remained unassociated with TMD (Supplementary Table S1). No evidence of horizontal pleiotropy was identified. Supplementary Figure S1 display leave-one-out plots, funnel plots, and scatter plots.

**FIGURE 3:**
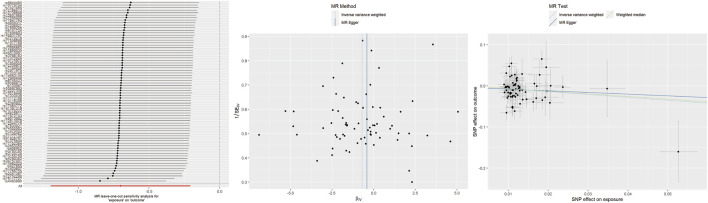
Leave-one-out plots, funnel plots, and scatter plots for getting up in morning as exposure and TMD as outcome.

For getting up in the morning, IVW and WM analyses indicated that genetically determined early risers had a decreased risk of TMD (IVW: OR = 0.50, 95% CI 0.30–0.81, *p* = 0.005; WM: OR = 0.52, 95% CI 0.29–0.96, *p* = 0.035). However, the causal estimate from MR-Egger did not reach statistical significance (OR = 0.66, 95% CI 0.09–4.64, *p* = 0.664). Unfortunately, heterogeneity was detected in heterogeneity tests. After removing the sources of heterogeneity (rs4483990, rs75117727), the results remained stable (Supplementary Table S1). No evidence of horizontal pleiotropy was found. Leave-one-out analysis suggested that excluding any individual SNP did not significantly alter or influence the overall evaluation conducted by the IVW method (Figure 3).

For snoring, all three analysis methods indicated that genetically determined snoring was not associated with the risk of TMD (IVW: OR = 3.47, 95% CI 0.73–16.37, *p* = 0.116; MR-Egger: OR = 0.55, 95% CI 0.01–88637.23, *p* = 0.924; WM: OR = 0.68, 95% CI 0.08–5.64, *p* = 0.724). No evidence of heterogeneity or horizontal pleiotropy was found. Leave-one-out plots, funnel plots, and scatter plots are provided in Supplementary Figure S2.

For daytime nap, all three analysis methods indicated that genetically determined daytime nap was not associated with the risk of TMD (IVW: OR = 1.08, 95% CI 0.63–1.85, *p* = 0.787; MR-Egger: OR = 1.64, 95% CI 0.23–11.76, *p* = 0.623; WM: OR = 1.28, 95% CI 0.65–2.55, *p* = 0.477). Heterogeneity was detected through heterogeneity tests. Upon removing the source of heterogeneity (rs1934138), daytime nap remained unrelated to TMD (Supplementary Table S1). No evidence of horizontal pleiotropy was found. Supplementary Figure S3 provide leave-one-out plots, funnel plots, and scatter plots.

### 3.3 Causal effect of TMD on sleep traits

When TMD was used as the exposure and sleep traits as the outcome, MR analysis results are presented in Table 2. MR analysis indicated that experiencing TMD does not result in changes in the five sleep traits. No evidence of heterogeneity or horizontal pleiotropy was detected. Supplementary Figures S4–S8 provide leave-one-out plots, funnel plots, and scatter plots.

**TABLE 2:** The causal effects of temporomandibular disorders on sleep traits.

Exposure	Outcome	nSNP	Methods	OR/Beta (95% CI)	*p*-value	Heterogeneity		Pleiotropy P-val
						MR-Egger Q_pval	IVW Q_pval	
TMD	Insomnia	14	IVW	0.99 (0.99–1.01)	0.278	0.815	0.859	0.712
		14	MR Egger	0.99 (0.98–1.01)	0.347			
		14	WM	0.99 (0.99–1.01)	0.240			
TMD	Sleep duration	14	IVW	0.01 (−0.01–0.01)	0.707	0.390	0.458	0.717
		14	MR Egger	−0.01 (-0.01–0.01)	0.956			
		14	WM	−0.01 (-0.01–0.01)	0.505			
TMD	Getting up in morning	14	IVW	1.00 (0.99–1.01)	0.512	0.618	0.682	0.691
		14	MR Egger	1.00 (0.99–1.01)	0.923			
		14	WM	1.00 (0.99–1.01)	0.922			
TMD	Snoring	14	IVW	1.00 (0.99–1.01)	0.849	0.058	0.065	0.490
		14	MR Egger	1.00 (0.99–1.01)	0.672			
		14	WM	1.00 (0.99–1.01)	0.351			
TMD	Daytime nap	14	IVW	1.00 (0.99–1.00)	0.250	0.260	0.273	0.407
		14	MR Egger	0.99 (0.98–1.00)	0.192			
		14	WM	0.99 (0.98–1.00)	0.127			

TMD, temporomandibular disorders; IVW, inverse variance weighted; WM, weighted median.

## 4 Discussion

To the best of our knowledge, the present study is the first investigation using MR analysis to examine the causal relationship between sleep traits and TMD at the genetic level. Leveraging comprehensive genomic data from over 400,000 European participants, we evaluated multiple exposure factors including insomnia, sleep duration, getting up in the morning, snoring, and daytime nap to infer potential causal associations between sleep traits and TMD. Our study findings indicate a significant negative correlation between getting up in the morning and TMD, suggesting that getting up in the morning serve as a protective factor against TMD. Additionally, insomnia may be considered a risk factor for TMD. However, our analysis did not find evidence supporting an influence of other sleep traits on the increased risk of developing TMD, nor did we observe that suffering from TMD directly leads to changes in sleep traits.

Sleep is crucial for restoring both mental and physical wellbeing. Sufficient and high-quality sleep can result in feeling refreshed and energized, and it is vital for sustaining cognitive function, normal physiological processes, and other essential life functions (Schütz et al., 2009). In contemporary society, sleep problems are widespread among the population, affecting approximately 30% of individuals who experience issues like insomnia, sleep deprivation, or interruptions (Roth, 2007). The quality of sleep is associated with psychological health, cardiovascular health, metabolic syndrome, and other related factors (Lian et al., 2019; Scott et al., 2021; Saz-Lara et al., 2022). The significance of sleep in the initiation and progression of diseases is also increasingly acknowledged. Sleep has been shown to be associated with various oral diseases. Reduced sleep duration and insomnia can elevate the risk of oral ulcers (Liu et al., 2023). Obstructive sleep apnea can elevate the risk of periodontitis (Mi et al., 2023).

While research on the connection between various sleep traits and TMD is limited, the association between sleep quality and TMD has been extensively investigated. Utilizing the Pittsburgh Sleep Quality Index for assessing sleep quality, studies have revealed that the sleep quality of TMD patients is significantly inferior to that of the control group (Rener-Sitar et al., 2016; Benoliel et al., 2017; Natu et al., 2018). Additionally, it has been observed that the severity of TMD is inversely proportional to sleep quality (Zamani et al., 2019; Yap et al., 2021). Nevertheless, the majority of prior studies have been cross-sectional, and due to methodological limitations, they are unable to establish a causal relationship between TMD and sleep.

Yap’s research revealed that within TMD patients, higher levels of pain correlate with poorer sleep quality. Nevertheless, irrespective of pain, TMD patients experience diminished sleep quality compared to individuals without TMD (Yap et al., 2021). While it is commonly believed that TMD-induced pain affects sleep quality, exploring the reasons for decreased sleep quality in TMD patients without pain is warranted. Furthermore, a longitudinal study revealed that prior to the onset of TMD, sleep quality had been progressively declining, and individuals with poor sleep quality had twice the risk of developing TMD compared to those with good sleep quality (HR: 2.04, 95% CI: 1.55–2.70) (Sanders et al., 2016). This is consistent with the conclusion of this study, indicating that individuals with insomnia have an elevated risk of developing TMD, while TMD does not cause insomnia. Insomnia may elevate the risk of TMD due to its association with psychological health issues such as anxiety and depression (Palagini et al., 2022), which are directly linked to an increased risk of TMD (Liou et al., 2023).

Additionally, our MR analysis revealed that maintaining a healthy habit of early rising acts as a protective factor against TMD. Previous studies have demonstrated that adolescents experiencing difficulty waking up in the morning face a 1.7 times higher risk of developing TMD compared to the control group (Fernandes et al., 2022). Additionally, a cross-sectional study of 204 college students found that individuals who habitually wake up early exhibit a lower prevalence of TMD compared to those who wake up late (Dan et al., 2024). Our study confirms the presence of this causal relationship. This could be because early risers generally lead healthier lifestyles (Plekhanova et al., 2023). Studies indicate that early risers typically have better mental health (Li et al., 2023), which is correlated with a lower incidence of TMD (Yeung et al., 2017). Furthermore, difficulty in waking up early is often due to late-night habits. Individuals who stay up late typically experience poorer sleep quality, which can contribute to the development of TMD (Barclay et al., 2010). From a molecular mechanism perspective, early sleepers and risers exhibit lower levels of inflammatory markers (Zhai et al., 2021), which helps reduce the risk of developing TMD. However, the pathophysiological mechanisms linking early rising and TMD remain to be fully studied. Further research is required to fully elucidate the complex relationship between early rising and TMD, along with the potential underlying mechanisms.

This study did not establish a causal relationship between sleep duration, snoring, daytime napping and TMD. However, sleep duration still affects the prognosis of TMD. Patients with TMD who have longer sleep duration exhibit improved treatment outcomes after 3 months compared to those with shorter sleep duration, possibly because insufficient sleep duration induces systemic inflammation (Kim et al., 2023). Currently, there is a lack of research investigating the association between snoring and TMD. Nevertheless, snoring frequently coexists with sleep apnea symptoms, and sleep apnea symptoms are a risk factor for TMD (Sanders et al., 2013; Al-Jewair et al., 2016). Consequently, it remains important to exercise caution regarding the potential impact of sleep apnea symptoms accompanying snoring on TMD.

Prevention of TMD is paramount as it circumvents additional time and financial burdens associated with treatment. Individuals grappling with insomnia and struggling to wake up early should exercise caution to prevent the onset of TMD. For those enduring chronic insomnia and frequently waking up late, the emergence of symptoms like jaw clicking or popping, along with jaw pain, warrants prompt consideration for TMD. When dentists encounter patients complaining of temporomandibular joint discomfort, it is crucial to assess their sleep condition. For patients already diagnosed with TMD, investigating potential treatments involving improved sleep therapy and habits may be beneficial. Simultaneously, promoting interdisciplinary collaboration between dentists and sleep specialists can enhance the comprehensive care of patients.

Our research has several advantages. Firstly, this study represents the inaugural bidirectional MR investigation into the causal relationship between five sleep traits and TMD. Secondly, diverse MR analysis methods were employed to validate the accuracy and robustness of the findings, and sensitivity analyses including MR-PRESSO were conducted to ensure consistent estimations of the causal effects in MR. Thirdly, the database utilized in this study was the most up-to-date and reliable. Nonetheless, there are several limitations that warrant attention. Firstly, the summary GWAS data were restricted to individuals of European descent, potentially introducing ethnic influence on causality. Secondly, the presence of heterogeneity in certain results could introduce biases into the estimates of causal effects. Heterogeneity was evaluated via leave-one-out analysis, demonstrating that the overall risk estimation remained largely unaffected.

## 5 Conclusion

This study, for the first time, examined the causal relationship between five sleep traits and TMD employing MR. Findings suggest that getting up in the morning is protective against TMD, whereas insomnia may constitute a risk factor. Furthermore, no causal association was found between sleep duration, snoring, daytime napping, and TMD; likewise, experiencing TMD does not influence sleep traits. Individuals with chronic insomnia and frequent late awakenings should be promptly evaluated for TMD if symptoms such as jaw clicking or popping, as well as jaw pain.

## Data Availability

The original contributions presented in the study are included in the article/Supplementary Material, further inquiries can be directed to the corresponding author.

## Abbreviations

GWAS, Genome-wide association study; IVs, Instrumental variables; IVW, Inverse variance weighted; MR, Mendelian randomization; SNPs, Single Nucleotide Polymorphisms; TMD, Temporomandibular disorders; WM, Weighted median.
