# Supplementary material for: Causal associations between sleep traits and temporomandibular disorders: a bidirectional mendelian randomization analysis
Source: Front Genet. 2024 Jul 24;15:1429190. doi: 10.3389/fgene.2024.1429190 (PMC11303139; doi:10.3389/fgene.2024.1429190)
Supplement: Supplementary file 2 [file Table1.DOCX]

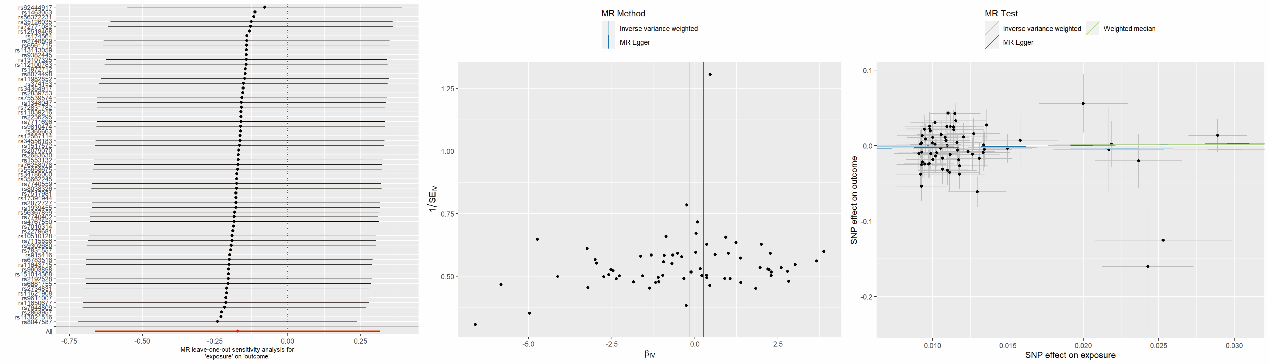


**Supplementary Figures 1** Leave-one-out plots,funnel plots, and scatter plots for sleep duration as exposure and TMD as outcome.


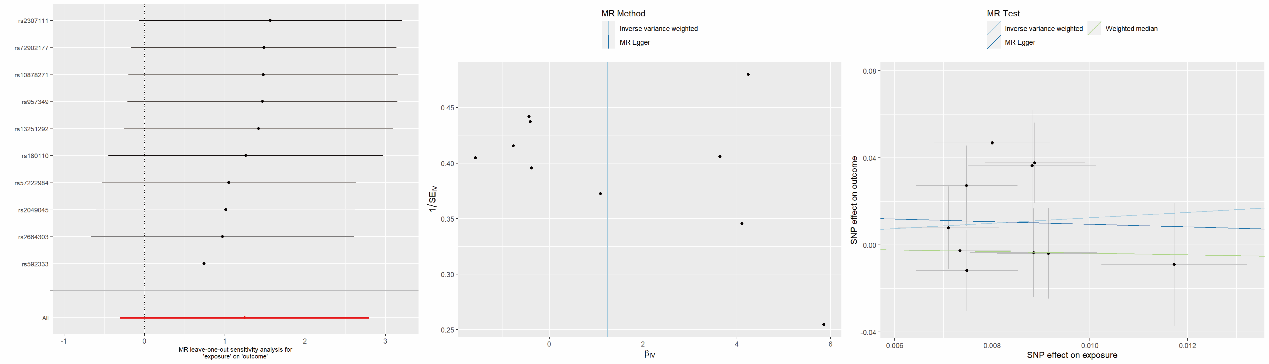


**Supplementary Figures 2** Leave-one-out plots,funnel plots, and scatter plots for snoring as exposure and TMD as outcome.


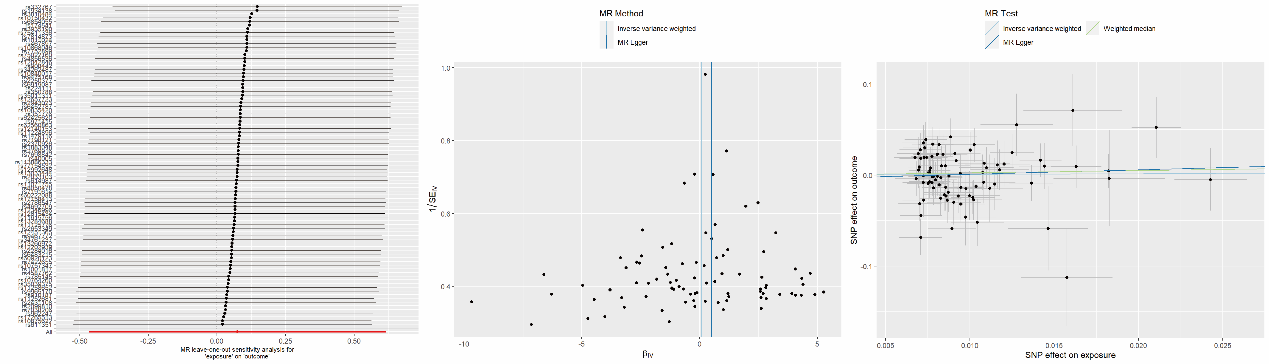


**Supplementary Figures 3** Leave-one-out plots,funnel plots, and scatter plots for daytime nap as exposure and TMD as outcome.


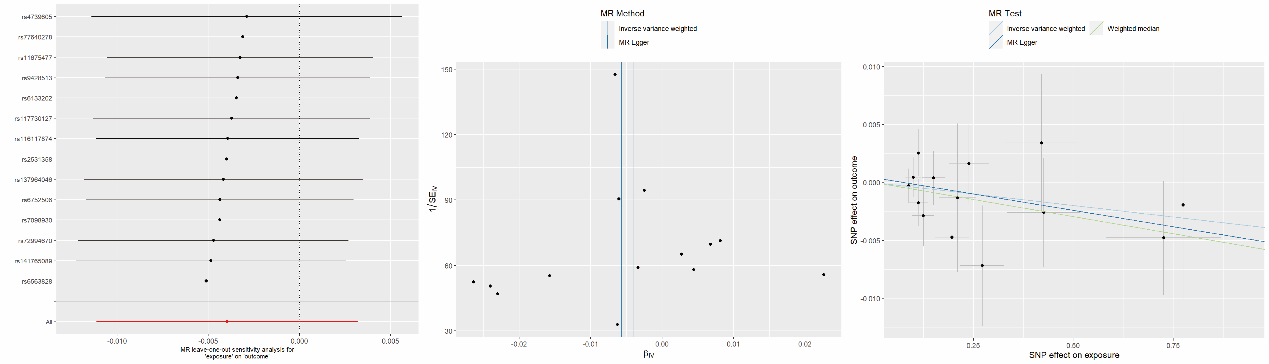


**Supplementary Figures 4** Leave-one-out plots,funnel plots, and scatter plots for TMD as exposure and insomnia as outcome.


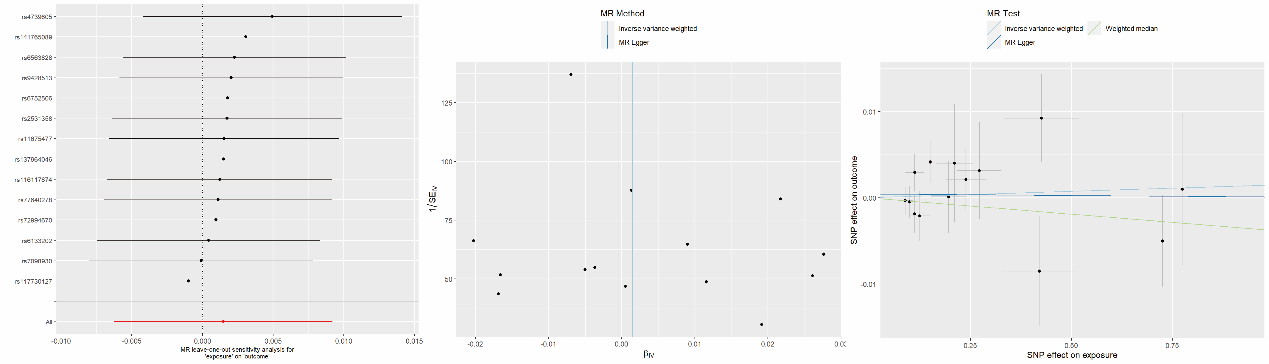


**Supplementary Figures 5** Leave-one-out plots,funnel plots, and scatter plots for TMD as exposure and sleep duration as outcome.


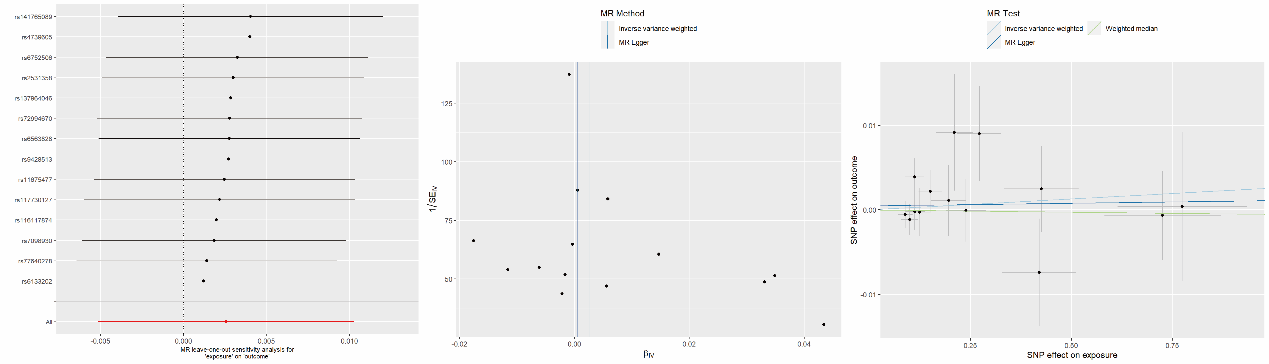


**Supplementary Figures 6** Leave-one-out plots,funnel plots, and scatter plots for TMD as exposure and getting up in morning as outcome.


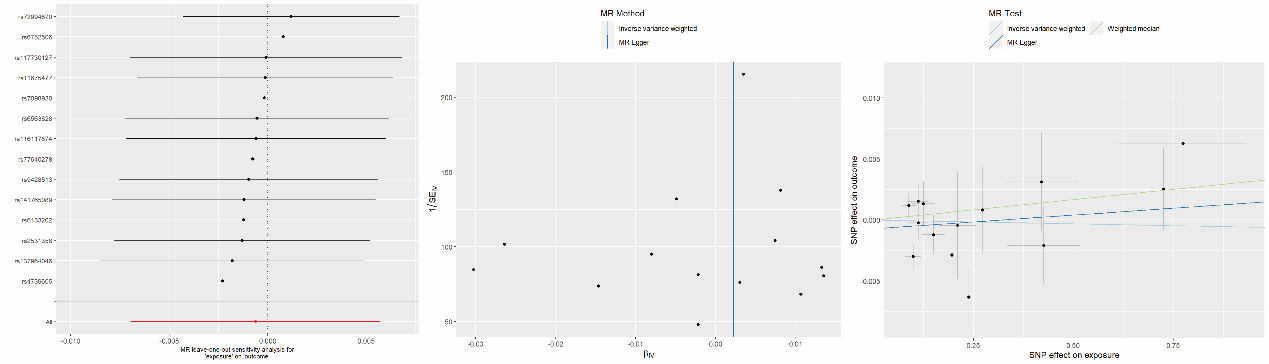


**Supplementary Figures 7** Leave-one-out plots,funnel plots, and scatter plots for TMD as exposure and snoring as outcome.


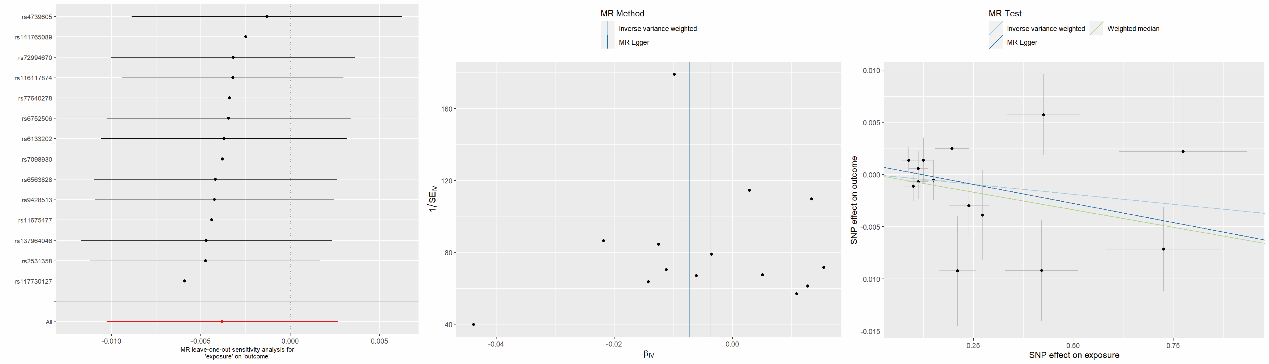


**Supplementary Figures 8** Leave-one-out plots,funnel plots, and scatter plots for TMD as exposure and daytime nap as outcome.
