# Supplementary material for: Causal associations between sleep traits and temporomandibular disorders: a bidirectional mendelian randomization analysis
Source: Front Genet. 2024 Jul 24;15:1429190. doi: 10.3389/fgene.2024.1429190 (PMC11303139; doi:10.3389/fgene.2024.1429190)
Supplement: Supplementary file 3 [file Table3.DOCX]

**Supplementary Table 1** The causal effects of sleep traits on temporomandibular disorders without outliers.

| **Exposure** | **Outcome** | **nSNP** | **Methods** | **OR**  **(95% CI)** | **P-value** | **Heterogeneity** | | **Pleiotropy**  **P-val** |
| --- | --- | --- | --- | --- | --- | --- | --- | --- |
|  |  |  |  |  |  | **MR-Egger**  **Q_pval** | **IVW**  **Q_pval** |  |
| Sleep duration | TMD | 66 | IVW | 0.92 (0.58-1.48) | 0.741 | 0.119 | 0.130 | 0.561 |
|  |  | 66 | MR Egger | 1.58 (0.24-10.11) | 0.632 |  |  |  |
|  |  | 66 | WM | 1.07 (0.56-2.07) | 0.826 |  |  |  |
| Getting up in morning | TMD | 70 | IVW | 0.39 (0.25-0.61) | **3.02E-05** | 0.243 | 0.270 | 0.902 |
|  |  | 70 | MR Egger | 0.35 (0.06-2.03) | 0.246 |  |  |  |
|  |  | 70 | WM | 0.51 (0.27-0.96) | **0.038** |  |  |  |
| Daytime nap | TMD | 86 | IVW | 1.16 (0.69-1.94) | 0.580 | 0.072 | 0.083 | 0.928 |
|  |  | 86 | MR Egger | 1.26 (0.19-8.27) | 0.811 |  |  |  |
|  |  | 86 | WM | 1.29 (0.63-2.63) | 0.492 |  |  |  |

TMD: Temporomandibular disorders; IVW: Inverse variance weighted; WM: Weighted median.
